# Interactive Video Game Cycling Leads to Higher Energy Expenditure and Is More Enjoyable than Conventional Exercise in Adults

**DOI:** 10.1371/journal.pone.0118470

**Published:** 2015-03-04

**Authors:** Javier Monedero, Elizabeth J. Lyons, Donal J. O’Gorman

**Affiliations:** 1 School of Health and Human Performance, Dublin City University, Dublin, Ireland; 2 University of Texas Medical Branch at Galveston, Institute for Translational Sciences, Galveston, Texas, United States of America; 3 Centre for Preventive Medicine, Dublin City University, Dublin, Ireland; University of the Balearic Islands, SPAIN

## Abstract

**Background:**

Despite the widely accepted health benefits of regular physical activity, only a small percentage of the population meets the current recommendations. The reasons include a wide use of technology and a lack of enjoyment while exercising. The purpose of this study was to compare the physiological, perceptual and enjoyment responses between a single bout of (I) conventional cycling and (II) interactive cycling video game at a matched workload.

**Methods:**

A cross-sectional study in 34 healthy participants was performed. Initially, participants completed an incremental maximal cycling test to measure peak oxygen uptake and to determine ventilatory threshold. In random order, participants carried out a 30 min interactive cycling trial and a 30 min conventional cycling trial at 55% of peak power output. During the trials, oxygen uptake and energy expenditure were measured by open-circuit spirometry and heart rate was measured by radiotelemetry. RPE and enjoyment were measured every 10 minutes with Borg scale and a modified PACES scale.

**Results:**

Interactive cycling resulted in a significantly greater %V̇O_2_Reserve (68.2% ± 9.2% vs 64.7% ± 8.1%), rate of energy expenditure (505.8±75.2 vs 487.4±81.2 j·kg^-1^·min^-1^), and enjoyment (63.4% ± 17 vs 42% ± 13.6), P&lt;0.05. Participants were working at a higher intensity in relation to the individual’s ventilatory threshold during the interactive cycling video game trial (*M* = 11.86, *SE* = 3.08) than during the Conventional cycling trial (*M* = 7.55, *SE* = 3.16, *t(33)* = -2.69, *P*&lt;0.05, r = .42). No significant differences were found for heart rate reserve (72.5 ± 10.4 vs 71.4±10.1%) and RPE (13.1 ± 1.8 vs 13.2 ± 1.7).

**Conclusion:**

Interactive cycling games can be a valid alternative to conventional exercise as they result in a higher exercise intensity than conventional cycling and a distraction from aversive cognitive and physiological states at and above the ventilatory threshold.

## Introduction

Regular physical activity plays an important role in the prevention of more than 25 chronic diseases [1,2,3], including cardiovascular disease, type 2 diabetes and some types of cancers [4]. Despite the known benefits of physical activity most people do not meet the minimum recommendations [5]. One of the main reasons cited is that advances in technology have decreased occupational and recreational physical activity [6]. The consequences include reduced daily energy expenditure and increased sedentary time which has recently been identified as an independent risk factor for chronic diseases [7]. Population based interventions and policies to promote PA have had limited success and this may partly be related to a lack of enjoyment when participating in traditional aerobic and resistance training programmes.

It is been reported that 40 to 65% of individuals drop out of a physical activity program within 3–6 months [8,9]. Physical activity is a complex behaviour associated with multiple correlates [10,11], including enjoyment in adults [11,12], which promote adherence [13,14,15,16]. Video game play is enjoyed by 58% of adult Americans [17] because the additional elements of fantasy, challenge and curiosity result in a more immersive experience than other games [18]. Active video games (AVGs) are a new type of video game that combine movement with video games and offer a promising strategy to increase PA adherence in the adult population.

In line with the behavioural choice theory, substituting conventional exercise with an activity that involves exercise but that is more enjoyable, in the form of AVGs, resulted in higher adherence rates and affect states [19,20]. The available evidence suggests that very light-to-vigorous physical activity can be achieved while playing AVGs [21,22,23,24,25,26]. There is also preliminary evidence that a short bout of interactive cycling video game (ICVG) results in higher energy expenditure (EE) and a similar rate of perceived exertion (RPE) than a similar bout of conventional cycling [23]. One of the challenges in AVG research is using comparable exercise modalities when comparing AVGs with conventional exercise. The GameBike is an ICVG that allows the player to control the movement of a vehicle displayed on a video screen by pedalling and using the handlebars.

The purpose of this study was to compare the physiological responses and enjoyment levels during a bout of ICVG and a similar bout of conventional stationary cycling (CSC) at the same workload. We hypothesised that ICVG would result in higher cardiovascular and metabolic response than a bout of CSC at a matched workload, but RPE would be lower while enjoyment ratings would be higher.

## Materials and Methods

### Participants

A total of 34 asymptomatic, young, men and women volunteered to take part in this study. All participants completed a Physical Activity Readiness Questionnaire (PAR-Q), and a general health questionnaire that included questions on PA participation in the last 6 months. The inclusion criteria for the study were as follows: 1) healthy male or female age 18–45 years, 2) non-smoker, 3) no more than one exercise session per week over the previous 6 months, 4) had a V̇O_2_peak within the 60^th^ percentile for the age and gender group. Participants refrained from drinking caffeine and alcohol for 24 h and from the ingestion of food or fluids (except water) for 3 hours before each test. The study complies with the principles of the declaration of Helsinki, was approved by the Dublin City University Research Ethics Committee and all participants provided written informed consent. Body mass index (BMI) was calculated from height, measured to the nearest 0.1 cm, and weight, to the nearest 0.1 kg (Seca Ltd, model 778, Germany). Given that the level of AVG playing experience varied considerably amongst participants, familiarisation sessions were provided. All testing sessions took place at the High Performance Laboratory at the School of Health and Human Performance at Dublin City University.

### Experimental overview

**Fig 1:**
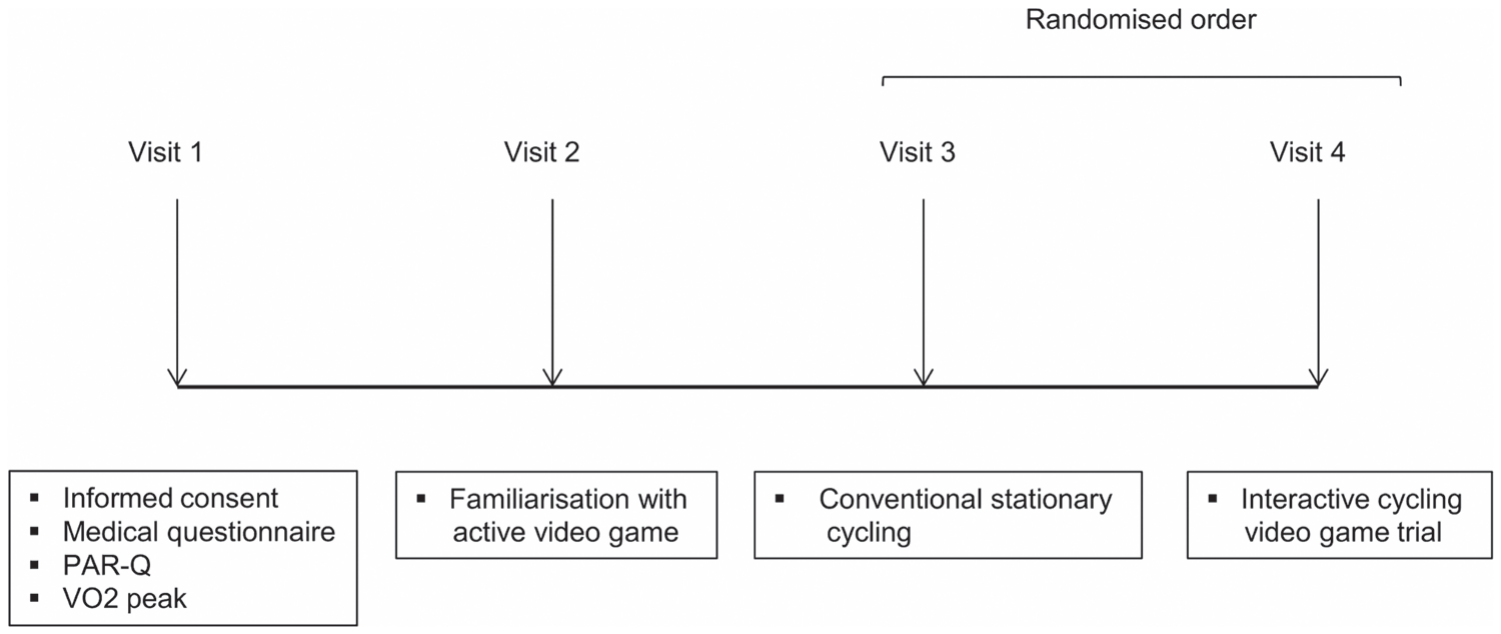
Schematic of study protocol.

Participants reported to the laboratory at Dublin City University on 4 different days separated by at least 48 h (Fig. 1). On day 1, participants carried out an incremental maximal test on a cycle ergometer (Velotron; RacerMate, Seattle, WA) to determine peak oxygen uptake (V̇O_2_peak) and peak power output. On day 2, participants familiarised themselves with the GameBike and the video game during a 30-min session. On the final two visits participants played the ICVG or cycled for 30-mins in random order.

### Peak Oxygen uptake test

V̇O_2_peak was determined by open-circuit spirometry (Innocor, Inn 00200, Innovision, Odense, Denmark). The protocol consisted of an initial power output of 50W and 75W for females and males respectively, 1 min duration with 15 W increments until exhaustion and at a self-selected cadence. V̇O_2_ was considered to have peaked if two of the following criteria were met: (i) a levelling off of V̇O_2_ with increasing power output (an increase of less than 2 mL·kg^-1^·min^-1^); (ii) a HR within 10 beats of the age predicted HRmax (220 beats·min^-1^—age in years); (iii) a RER greater than 1.10. The peak power output was defined as the highest power output in watts (w) that participants were able to reach. We determined this by using the following equation [27]:

$$Peak\;power\;output\;\left(w\right)\;=\;POfinal+\left(\frac{t}{60}\right)15$$ (1)

where POfinal = the last exercise intensity completed for 1 min (W) and t = the number of seconds for which the final, uncompleted exercise intensity was sustained, and 15 was the PO increment in watts. After the test was completed, the ventilatory threshold was determined using the V-slope method [28] by two independent experts.

### Conventional stationary cycling and interactive video game cycling trials

For the exercise and gaming trials, participants reported to the laboratory and were lying in a supine position for 10 min while physiological variables were recorded. The last 3 min of these 10 min were averaged to calculate baseline values for oxygen consumption and heart rate. Following this, participants moved to the cycle ergometer and exercised or played the ICVG for 30 min at a matched workload of 55% of peak power output and then remained seated in a chair for 20 min. The electronically braked cycle ergometer (Velotron; RacerMate, Seattle, WA) was modified as described elsewhere in the literature [23] to be able to adapt the handlebar and speed sensor from a GameBike GB 200 ICVG (CatEye, Osaka, Japan) and interface it with a Sony Playstation 2 (Sony, San Mateo, CA). The video game requires the player to pedal in order to move a vehicle displayed on a video screen. The handlebars on the bicycle ergometer allow the vehicle to move left or right while travelling around a race track. The video game provides visual and audio feedback, including music and mechanical sounds. All participants played the Playstation 2, Road Fury 2 game and cycled on the same track and terrain, using the same bike and game setting (Single Event, Hampworth Station) while competing against 5 computer riders.

### Physiological measurements

Physiological variables measured included V̇O_2_, carbon dioxide production (VCO_2_), respiratory exchange ratio (RER), minute ventilation (V_E_), metabolic equivalents of the task (METs), rate of energy expenditure (rate EE) and HR. These measurements were performed while in supine position before each trial, during the exercise/active gaming part of the trial and for 20-mins after each trial. Following completion of the test, the % V̇O_2_peak above or below the VT was calculated.

The rate of EE was estimated using the Consolazio et al. [29] formula and converted to j·kg^-1^·min^-1^:

Rate of EE = (3.78×VO2)+(1.16×VCO2) in kcal/min (2)

The percentage HR reserve (%HRR) was calculated by using a derivation of the Karvonen et al. formula [30]:

$$\%\;HRR\;=\;\frac{HRtrial-RHR}{HRmax-RHR}\times 100$$ (3)

where HRtrial = average HR during the trial, RHR = resting HR and HRmax = HR max (as determined during a V̇O_2_peak test). We calculated the % V̇O_2_peak above or below the VT to get an indication of the metabolic intensity of the trials by using the following formula:

$$\%VT\;=\;\frac{\left(VO2trial-VO2@VT\right)\times 100}{VO2@VT}$$ (4)

where % VT = % V̇O_2_peak above or below the V̇O_2_@ VT, V̇O_2_trial = average oxygen uptake during the trial in L/min, V̇O_2_@VT = oxygen uptake at VT in L/min.

### Affect and psychological measurement

The research participants filled in a modified PACES scale to assess enjoyment after 10, 20 and 30 min of each trial. The modified PACES scale contained 6 of the original 18 bipolar statements with 7 points between statements. The responses were added to give a score ranging from 6 to 42 for each activity and a percentage enjoyment result was calculated. The PACES scale is a reliable and valid measure of enjoyment in physical activity environments [26,31,32,33]. Reliability of the 6 items was high for each activity using pooled data from the 2 gender groups (Cronbach’s alphas ≥ 0.942). We used the 15 point Borg scale [34] to determine participants’ level of perceived exertion.

### Statistical analysis

All figures and tabular values are reported as the mean ± SD. IBM SPSS v.19 (SPSS, Inc. USA) was used to analyse the data and the level of significance was set at *P* < 0.05. All data were tested for normal distribution with the Shapiro-Wilk test. Sphericity was assessed for each of the variables and the Greenhouse and Geisser’s correction for the degrees of freedom was applied when sphericity was not met. To investigate differences within subjects by trial and by time within each trial, a mixed design ANOVA was performed. Gender was included as a between subjects variable. Multiple comparison tests were corrected using the Bonferroni method. For a comparison of overall means a paired t-tests was used. For those variables that did not meet parametric statistic assumptions (enjoyment and RPE), a Wilcoxon signed rank test was used. An unpaired t-test was used to detect significant differences between subject characteristics. The relationship between enjoyment and RPE was determined by Pearson’s product correlation coefficient.

## Results

### Participant characteristics

The physical characteristics of the participants are summarised in Table 1. There were some expected differences between males and females for height, aerobic fitness and peak power output. The males were also significantly older than females.

**Table 1:** Physical characteristics and cardiorespiratory responses during maximal bike test.

	Male (n = 18)	Female (n = 16)	All (= 34)
**Age (yr)**	28.1±7.7*	22.6±4.6	25.5±6.9
**Height (m)**	1.76±0.0*	1.66±0.1	1.72±0.1
**Body mass (kg)**	78.5±8.5*	66.1±9.7	72.6±10.9
**BMI (kg**·**m** ^**-2**^ **)**	25.3±3.1	23.9±3.4	24.6±3.3
**Resting HR (b**·**min** ^**-1**^ **)**	62.7±10.0*	72.9±13.2	67.5±12.5
**HRmax (b**·**min** ^**-1**^ **)**	185.9±9.0*	189.8±14.0	187.7±11.6
**V̇O**_**2**_ **peak (mL**·**kg** ^**-1**^·**min** ^**-1**^ **)**	39.9±5.2*	29.9±5.2	35.2±7.2
**% V̇O**_**2**_ **peak @ VT**	63.0±9.1	66.4±10.7	64.6±9.9
**Peak PO (W)**	263.3±36.0*	174.7±26.6	221.6±54.8
**55% Peak PO (w)**	143.9±20. 7*	96.1±14.6	121.5±130

Data presented as Mean ± SD.

* significantly different (*P* < 0.05). HRmax: maximal heart rate, V̇O_2_peak (mL·kg^-1^·min^-1^): rate of maximal oxygen uptake (milliliters·kilograms^-1^·minute^-1^), Peak PO (W): peak power output in watts.

### Physiological variables

There were no statistically significant differences in resting V̇O_2_, HR and the rate of EE between CSC and ICVG. The female participants had a higher HR response than males participants (*P* ˂ 0.001), and males had a higher V̇O_2_, and rate of EE (*P* ˂ 0.001). There were no interactions between sex and trial or time. Even though the workload was identical in both trials the % V̇O_2_peak was significantly higher during the 30 min ICVG (*M =* 71.06%, *SE =* 1.34) compared with CSC (*M =* 68.48%, *SE =* 1.26, *t*(33) = -2.48, *P*<0.05), *r =*. 39), see table 2. Comparisons at intermittent time points reveal significantly higher values during ICVG after 10-mins (*M =* 69.12%, *SE =* 1.40) than during CSC (*M =* 65.28%, *SE =* 1.25, *t*(33) = -3.0, *P*<0.05), *r =*. 53) but not the other time points. The percentage of HRmax increased significantly over time in both trials (*P* < 0.01, partial η^2^ = .777) but there were no significant differences in %HRmax or heart rate reserve between trials.

**Table 2:** Physiological responses to CSC and ICVG.

	CSC	ICVG
**HRmax (%)**	81.5±6.7	82.5±7.0
**HRR (%)**	71.5±10.1	72.7±10.1
**V̇O2peak (%)**	68.5±7.4	71.1±7.8*
**V̇O**_**2**_ **R (%)**	64.7±8.1	68.2±9.2*
**Rate of EE (j**·**kg** ^**-1**^·**min** ^**-1**^ **)**	487.4±81.2	505.8±75.2*
**VT (%)**	7.6±17.9	11.9±18.4*
**Cadence(rpm)**	84.9±17.7	85.4±23.5

Data are presented as, Mean ± SD, (n = 34). HR: heart rate, % HRmax: percentage maximal heart rate, HRR(%): percentage heart rate reserve, Rate of EE (j·kg^-1^·min^-1^): rate of energy expenditure, V̇O_2_: oxygen uptake, V̇O_2_R:oxygen uptake reserve, % VT: percentage above the ventilatory threshold.

*: Significantly different (*P* < 0.05).

A significant main effect of trial on the rate of EE *F*(1,32) = 5.610 (*P* < 0.05, partial η^2^ = .149) was found. Paired-t-tests revealed that participants had a higher rate of EE during the ICVG (*M* = 505.8 j·kg^-1^·min^-1^, *SE* = 12.9) than during the CSC trial (*M =* 487.4 j·kg^-1^·min^-1^, *SE =* 13.9, *t(33)* = -2.44, *P*<0.05, r = .39). These differences were also significantly different at 0–10min (*P* = 0.002) and approached statistical significance at 10–20min (*P* = 0.07). The rate of EE increased over time in both conditions (*P* < 0.01), partial η^2^ = .697).

In order to evaluate the metabolic intensity of each trial, the ventilatory threshold was calculated for each participant and compared with their %VO_2_peak during each trial. The %V̇O_2_ peak above the ventilatory threshold was significantly higher during the ICVG trial (*M* = 11.86, *SE* = 3.08 vs. *M =* 7.55, *SE =* 3.16, *t(33)* = -2.69, for the CSC trial, *P*<0.05, r = 0.42).

**Fig 2:**
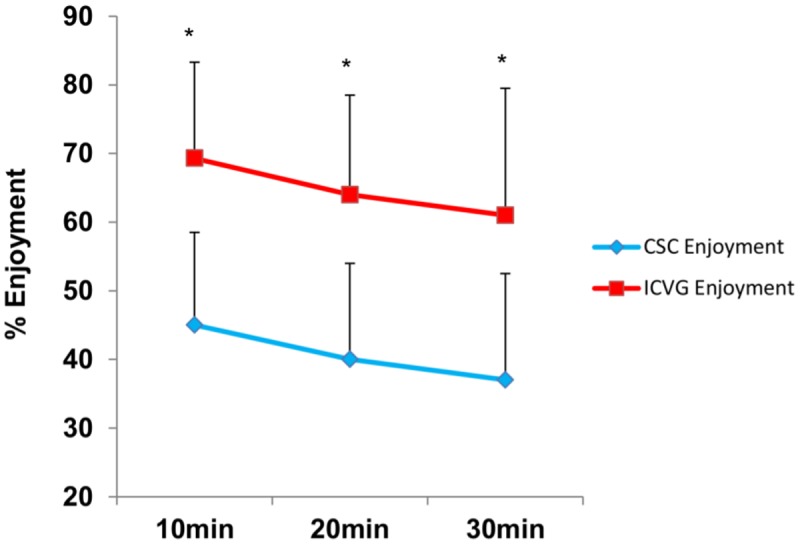
Enjoyment and RPE scores during CSC and ICVG trials. Data are presented as, mean ± SD, (n = 34). * Significant difference (*P* < 0.01).

**Fig 3:**
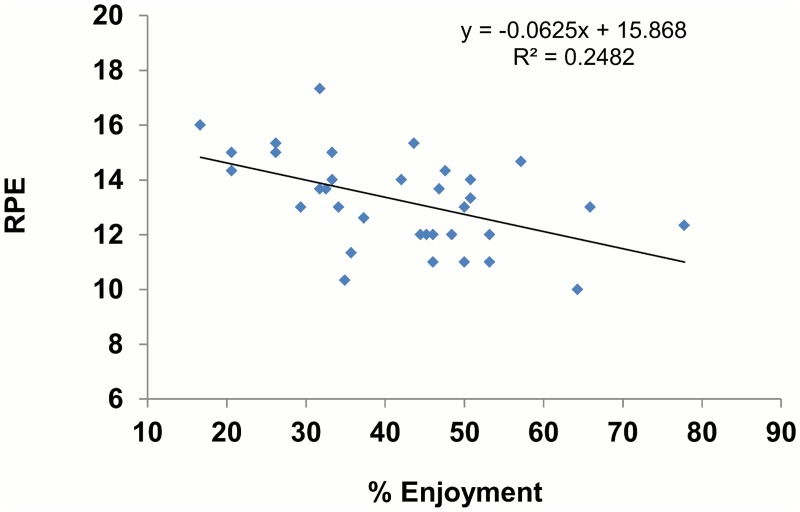
Relation between RPE and enjoyment in CSC trial.

**Fig 4:**
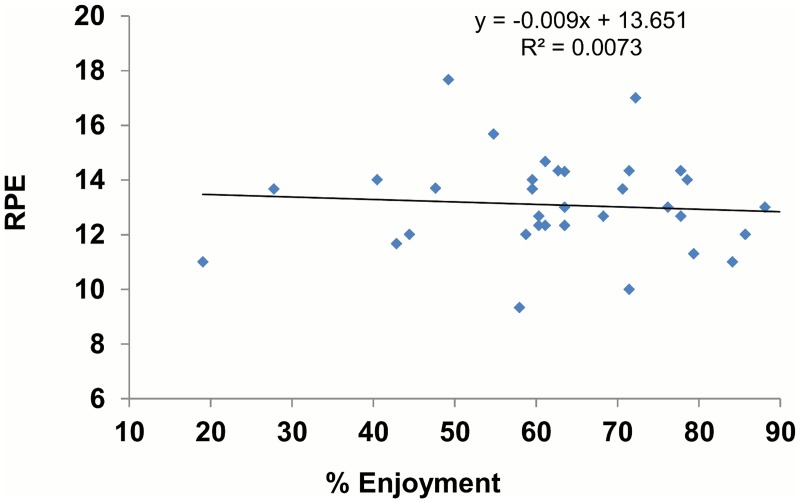
Relation between RPE and enjoyment in ICVG trial.

The mixed design ANOVA revealed a significant effect of trial (*P* < 0.001, partial η^2^ = .680) and time (*P* < 0.001, partial η^2^ = .499) on enjoyment (see Fig. 2). During the ICVG trial participants reported greater levels of enjoyment than the CSC trial (*M* = 23.95, SE = 2.90, *P* <. 001). Mean enjoyment decreased similarly over time in both trials, with significant differences from 10 to 20 minutes (*M* = 5.51, SE = 1.10, *P* < 0.001) and 20 to 30 minutes (*M* = 2.87, *SE* = 1.09, *P* = 0.038). When overall ratings were compared in the two trials, enjoyment was higher in the ICVG than in the CSC (*t* = 6.21, *P* < 0.001). We also report a significant effect of gender in the enjoyment responses (*P* = 0.046), as men had higher levels of enjoyment across trial and time periods (Table 3). The rating of perceived exertion increased at each 10-min interval (*P* < 0.001, partial η^2^ = 0.837) but there was no effect of trial or gender (*P* > 0.05). We report a negative correlation between RPE and enjoyment in the CSC trial (r = -0.498, *P*<0.01), but this relation was not present in the ICVG trial (Figs. 3 and 4). Further analysis revealed that RPE accounted for 24.8% of the variance in enjoyment during the CSC trial.

**Table 3:** Psychological and affect responses to CSC and ICVG.

	CSC			ICVG		
	Male	Female	Total	Male	Female	Total
**RPE**						
**10 min**	11.3±1.8	11.4±1.6	11.4±1.7	10.6±1.4	11.6±2.1	11.1±1.8
**20 min**	13.2±1.8	14±1.9	13.6±1.8	12.6±1.6	14.2±1.6	13.3±1.8
**30 min**	14.3±1.8	15.4±2.4	14.8±2.2	13.9±1.8	15.9±2.2	14.8±2.2
**Average**	12.9±1.6	13.6±1.8	13.2±1.7	12.4±1.4	13.9±1.8	13.1±1.8
**Enjoyment (%)**						
**10 min**	46.8±15.8^b^	43±10.3	45±13.5	73±16^a^ ^,^ ^b^	65.2±11^a^	69.3±14^a^
**20 min**	43.8±14.5^b^	35.7±12.5	40±14	66.7±17^a^ ^,^ ^b^	59.8±11.2^a^	64±14.5^a^
**30 min**	41.7±17.3^b^	31.4±11.4	37±15.5	65.5±18^a^ ^,^ ^b^	56±18^a^	61±18.5^a^
**Average**	44.1±15.2^b^	36.7±10.6	42±13.6	68.4±16^a^ ^,^ ^b^	60.3±11.8^a^	63.4±17^a^

Data are presented as mean ± SD, (n = 34).

a: Significantly higher than CSC (*P* <0.01).

b: Significantly higher than female for the same trial (*P* <0.05).

## Discussion

The main finding of this study was that a single bout of ICVG resulted in a significantly higher rate of EE than a bout of conventional cycling. Also, the ICVG was more enjoyable and led to a decrease in the negative affect states associated with high intensity exercise described in the literature [35]. Therefore, ICVG offers a viable alternative for adults to achieve the physical activity recommendations.

A number of studies have looked at the acute effect of adding a video game to stationary cycling in diverse populations [23,36,37]. Haddock *et al*. [36] reported a significantly higher difference in the net increase in energy expenditure during the ICVG than during CSC. Similar to our findings, Haddock *et al*. [36] did not find any significant differences in average HR and RPE in 7–14 year old children despite higher rates of EE during the ICVG than during the CSC. Warburton *et al*. [23] compared the metabolic demands of ICVG and CSC at 25%, 50% and 75% of peak power output. Participants exercised on two separate days at three incremental stages using a constant workload for 5 min with 5 min rest intervals. They reported significantly higher HR, V̇O_2_ and rates of EE at 25% and 50% of peak power output, during the ICVG than during CSC. While we have reported significant differences in V̇O_2_ and rate of EE, the differences in HR, V̇O_2_ and rate of EE at 25% and 55% reported by Warburton *et al*. [23] are higher than those reported in the present study. Our study builds on the findings of Warburton *et al*. (2009) as we used a longer protocol and measured enjoyment as an important parameter of the affect construct. We also found that participants were exercising at a significantly higher % V̇O_2_ peak relative to VT during the ICVG than during the CSC trial.

The higher rates of EE and higher V̇O_2_ during the ICVG trial are likely to be explained by different factors. Firstly, the ICVG trial could have provided distraction from the physical discomfort of exercising as it provided both visual and audio interaction. This is supported by the evidence that music has a beneficial effect on athletic performance and masks unpleasant feelings associated with intense exercise [38,39]. Secondly, the ICVG was likely to induce an increased state of arousal due to the visual and auditory stimulation while playing the interactive game. Thirdly, the participants were using their upper bodies to steer the handlebar during the game and this is likely to increase V̇O_2_ and energy demand due to increased muscle fibre recruitment. Both trials required upper body muscles for stability but steering was not possible in the conventional trial. However, both HR and cadence were similar between trials so it is unlikely that arousal caused the difference in the metabolic responses. This contrasts with the study by Warburton *et al*. [23] in which the ICVG resulted in 9% higher cadence than the CSC. It is possible that the game challenges in Warburton *et al*. [23] protocol, challenges by which the participants were required to cycle at different cadences throughout the game as part of the competition, led to the observed difference in HR and cadence.

One important finding in this study was that the ICVG trial resulted in significantly higher ratings of enjoyment than the CSC trial. These results are similar to those of short intervention studies comparing ICVG with conventional cycling [20,40] and other studies comparing AVGs with conventional exercise [41]. This may be due to the challenge presented in the video game condition and/or the visual and auditory stimulation. This challenge and sensory stimulation may have contributed to an increased state of flow in the ICVG. There is evidence that video games and sports are two domains in which the state of flow occurs [42,43,44], and this is considered to be very important in affective judgement [12]. We did not find any differences in RPE between trials, and these results are in agreement with those from other studies [23,36], but we did find a negative correlation between RPE and enjoyment in the CSC trial. This relationship was not evident in the ICVG trial, which suggests that the ICVG trial blunted the decrements in enjoyment usually associated with vigorous exercise intensity. This may have important public health implications as there is evidence supporting a positive impact of enjoyment on PA participation [13,14,15] and adherence [20].

This point is also emphasised by the finding that participants exercised at a higher intensity relative to their individual VT during the ICVG trial than during the CSC trial. Despite this, the ICVG trial resulted in enhanced affect states as the greater enjoyment rates shows and there were no differences in RPE. This finding seems to contradict one of the main tenets of the dual mode theory [45], that affective responses to exercise are influenced by the continuous interplay of cortically mediated cognitive processes (e.g., self-efficacy, self-presentational concerns, goals, attributions) and ascending interoceptive cues (e.g., ventilation, acidosis, core temperature). Positive affective states decrease as the VT stage is reached and passed [46]. Results from this study show that enjoyment levels were not negatively affected by a more stressful physiological state brought upon by high intensity exercise during gaming. It is possible that playing the active video game worked as an effective distraction from the ascending interoceptive cues associated with increasing exercise intensity. As a result the enjoyment ratings during the CSC trial were significantly lower than during the ICVG trial but participants were working at a significantly lower metabolic stress condition during the CSC trial.

The data presented in this paper provides strong evidence of the efficacy of active video game technology to address the problem of physical inactivity and increasing rates of sedentary behaviour. This data adds to the considerable body of evidence [20,23,24,25,26,47,48] supporting the inclusion of active video gaming in the repertoire of activities to increase population physical activity levels and reduce sedentary behaviour. While there are detractors and opponents of active video gaming, the data presented shows that it is possible to meet international physical activity guidelines and have a positive affect experience while doing this.

The implementation of applied studies, such as this, have several limitations. Firstly, the baseline value for the assessed physiological parameters was recorded as the average of the last 3 min of a 10 min supine rest. A longer supine rest would have been better to determine more accurate baseline HR, V̇O_2_ and rates of EE and these values may have been lower if a longer rest period had been allowed. This would have further strengthened the findings. Secondly, all testing took place in an artificial laboratory setting as opposed to a familiar home setting where active video game play usually takes place. Thirdly, while the exercise trials were performed at the same time of day the 3-hr lapse since last eating may have influenced the calculation of energy expenditure. Every effort was made to ensure the data was reliable while trying to maintain a ‘real life’ study design. Finally, the calculation of ventilatory threshold presents some methodological challenges and is prone to investigator bias. We minimised this bias by getting two experts to calculate the VT and using the function to calculate the VT available in the Innocor software in a supportive manner.

In conclusion, an acute bout of interactive cycling video game resulted in metabolic and cardiovascular responses that exceeds the international recommendation for physical activity and could be used to attain the public PA guidelines. The interactive cycling video game resulted in similar perceived effort and greater enjoyment ratings than a similar bout of conventional cycling. Interactive cycling video game is a valid alternative to conventional exercise since they result in higher exercise intensities than conventional cycling and a distraction from aversive cognitive and physiological states at and above the ventilatory threshold. The combination of conventional exercise and active video games in PA programs is a topic of research that should be explored to ascertain efficacy of both forms of exercise in the same program.
